# Perforated gastric carcinoma: a report of 10 cases and review of the literature

**DOI:** 10.1186/1477-7819-4-19

**Published:** 2006-03-30

**Authors:** Franco Roviello, Simone Rossi, Daniele Marrelli, Giovanni De Manzoni, Corrado Pedrazzani, Paolo Morgagni, Giovanni Corso, Enrico Pinto

**Affiliations:** 1 Dipartimento di Chirurgia Generale ed Oncologica, University of Siena, Italy; 2 Istituto di Semeiotica Chirurgica, University of Verona, Italy; 3 Divisione di Chirurgia 1, G.B. Morgagni Hospital, Forlì, Italy

## Abstract

**Background:**

Perforation is a rare complication of gastric carcinoma, accounting for less than 1% of all gastric cancer cases. The aim of the present study is to evaluate the prognostic value of perforation and to point out the surgical treatment options.

**Methods:**

A total of 10 patients with perforated gastric carcinoma were retrospectively reviewed among 2564 consecutive cases of gastric cancer operated in three Centers belonging to the Italian Research Group for Gastric Cancer. The clinicopathological features including tumor stage and survival were analyzed and compared to literature data.

**Results:**

Incidence rate was 0.39%. All patients underwent emergency surgery, being performed gastrectomy in 6 patients (mortality 17%) and repair surgery in 4 patients (mortality 75%). The survival of patients was related to the stage of the disease, with 2 long-survival cases.

**Conclusion:**

Perforation usually occurs in advanced stages of gastric cancer; nevertheless surgeons should not be always discouraged from a radical treatment of perforated gastric cancer, since perforation even occurs in early stages and seems not to be a negative prognostic factor itself. When possible, emergency gastrectomy should be performed, leaving repair surgery for unresectable tumors. A two-stage treatment is a good treatment option for frail patients with resectable tumors.

## Background

Perforation of gastric carcinoma results in an acute abdominal syndrome due to the spilled gastric contents and the consequent peritonitis. It is a rare condition representing less than 1% of gastric cancer cases in the reports of the last years[1,2] and up to 6% in reports dated before 1980 [3-5]; it has been reported that about 10–16% of all gastric perforations are caused by gastric carcinoma [6-9]. In most instances gastric carcinoma is not suspected as the cause of perforation prior to emergency laparotomy and the diagnosis of malignancy is often made only on postoperative pathologic examination. It is often difficult to recognize the kind of lesion that caused gastric perforation at the time of emergency surgery, particularly when pathologic evaluation of frozen sections is not available. The treatment should aim to manage both the emergency condition of peritonitis and the oncologic technical aspects of surgery: it may be hazardous to embark on a major procedure observing the principles of radical oncological surgery; on the other hand a limited procedure only may jeopardize long-term survival in a patient with potentially curable gastric malignancy. In order to further understand the optimal management of patients with perforated gastric cancer, we reviewed the clinicopathological features and surgical results in our experience, comparing data with the International literature.

## Methods

We reviewed the medical records of 2564 patients with gastric cancer who had undergone surgical treatment in three Centers belonging to the Italian Research Group for Gastric Cancer (IRGGC): Dipartimento di Chirurgia Generale ed Oncologica, University of Siena, Istituto di Semeiotica Chirurgica, University of Verona and Divisione di Chirurgia 1, G.B. Morgagni Hospital, Forlì. Ten patients (0.39%) were treated for perforated gastric carcinoma. The clinicopathological features of all patients were analyzed on the basis of their medical records. Age and sex, preoperative diagnosis, location of perforation, depth of gastric wall invasion, absence or presence of lymph node metastasis, type of surgery, degree of lymph node dissection, UICC stage and outcome of the patients were examined. Overall survival from the time of primary operation was calculated using Kaplan-Meier estimates. A search of the literature was conducted in the Medline database; the terms "perforated", "perforation", "gastric cancer", "gastric ulcer" were associated for the search and English language journals only were selected.

## Results

Clinicopathological features of patients are given in Table 1. The incidence rate of perforation among gastric carcinoma was 0.39%. Most cases were tumors invading serosa (4/6) and with metastatic lymph nodes (4/6). The disease was more frequently in stages III/IV (7/10), but one case (1/10) of stage I gastric cancer was also observed. All patients underwent emergency surgery. In only 3 patients on 10 a preoperative diagnosis of gastric carcinoma was made. Table 2 shows surgical and postsurgical survival data. Operations performed were gastrectomy in 6 patients and simple closure in 4 patients. Surgery-related deaths were observed in 4 patients: 3 of them underwent simple closure and 1 subtotal gastrectomy. All tumors treated with simple closure were at clinical stage IV of the disease and emergency gastrectomy was not performed because of the advanced stage with adjacent organs invasion. Five subtotal gastrectomies (4 D1 and 1 D2) and one D3 total gastrectomy were performed. Three surgical and two non-surgical complications were observed. The only patient who survived surgery after simple repair died at 5.2 months from operation for the primary disease. The only patient who underwent gastrectomy whose death was surgery-related was 80 and presented cardiologic comorbidity. Two patients underwent adjuvant chemotherapy and they both are still alive after 47.7 and 41.6 months after surgery, one with no evidence of disease and the other with bone recurrence.

**Table 1:** Clinicopathological features of patients with perforated gastric cancer.

**Variable**	**Number of Patients**
Age	
Range (yr)/Mean	50–82/68
Sex	
Male	6/10
Female	4/10
Preoperative diagnosis	
Perforation	10/10
Cancer	3/10
Location	
Lower third	8/10
Middle third	1/10
Upper third	1/10
Serosal invasion*	
Absent	4/6
Present	2/6
Lauren histological type*	
Diffuse	1/5
Intestinal	4/5
Lymph node metastasis*	
Absent	4/6
Present	2/6
Stage of disease	
I	1/10
II	2/10
III	3/10
IV	4/10
Surgery	
Gastrectomy	6/10
Local repair	4/10
Lymph node dissection	
Extended (D2, D3)	2/6
Limited (D0, D1)	4/6

*data not available for all patients.

**Table 2:** Postsurgical survival data for patients with perforated gastric carcinoma.

**Case**	**Sex**	**Age**	**TNM**	**Stage**	**Type of surgery**	**Comorbidities**	**Postoperative Complications**	**Survival (months)**	**Cause of death or Comments**
1	M	52	T4N1M0	III	DG-D1	Pulmonary	-	47,67	CHT – Alive with bone recurrence
2	F	82	T4N1M0	III	DG-D1	-	-	16,53	Primary cancer
3	M	76	-	IV	Repair	-	Pulmonary heart	<1	Surgery-related
4	F	78	T3N0MX	II	DG-D1	Cardiac	-	<1	Surgery-related
5	F	73	-	IV	Repair	-	Pulmonary embolism	<1	Surgery-related
6	M	81	T2N0MX	I	DG-D1	-	Anastomotic Leakage	18,80	Primary cancer
7	M	57	-	IV	Repair	-	-	<1	Surgery-related
8	F	65	-	IV	Repair	-	Bleeding	5,20	Primary cancer
9	M	66	T2N1MX	II	DG-D2	-	-	41,60	CHT – Alive
10	M	50	T3N2MX	III	TG-D3	-	Bleeding	25,60	Primary cancer

DG, distal gastrectomy; TG, total gastrectomy; D1, D2, D3, lymph node dissection; CHT, adjuvant chemotherapy.

## Discussion

Perforation is a rare complication of gastric cancer. In our series an incidence of less than 1% (0.39%) was observed comparable to the most recent studies[1,2]. Preoperative diagnosis of malignancy is unusual, accounting for about 30% of cases[1,2,10]; the other patients are usually accepted for acute abdomen at the Emergency Units where generic preoperative diagnosis of gastroduodenal perforation is made. The only preoperative feature that may guide the surgeon is the age of the patient: perforated gastric carcinoma usually occurs in patients with a mean age of 65 years (68 years in our series) in contrast with the mean age of 51 years of the patients with perforated peptic ulcers [9-13]. Even during surgery the gastric ulcer is often diffucult to be characterized as benign or malignant by the surgeon. Therefore a biopsy and frozen section should be performed in all gastric perforations when a pathologist is available. Histologic determination is fundamental for the surgeon to choose the type of operation and to perform it with oncological criteria, for example considering adequate distance from the lesion and the resection margin. Malignant gastric perforation is more often a manifestation of advanced cancer with serosal invasion (55–82%) and lymph node metastasis (57–67%). Nevertheless, as confirmed by different observations[14,15], gastric cancer can perforate at an early stage. Indeed at the pathologic examination of specimens, the process of gastric wall perforation is sustained by infectious and ischaemic factors due to the tumoral neovascularization which result in the shedding of the neoplastic tissue[3,16].

It is still debated whether positive peritoneal cytology has an independent prognostic impact in gastric cancer. Several studies have noted free gastric cancer cells in the peritoneum to be associated with poor prognosis[17,18]. However, viable free cancer cells have not been demonstrated in the peritoneal cavity of patients with perforated gastric cancer and the metastatic efficiency of gastric cancer cells possibly shed during perforation is uncertain in the presence of the peritonitis; different studies, included the present one, report of long-term survivors[19]. When a curative operation can be performed, survival rates after gastric cancer perforation[1,20] appear similar to survival rates observed in elective patients[21,22]. Moreover, Gertsch et al. demonstrated how the only factor predicting long term survival is the TNM stage, while age or the size, the location, the depth of infiltration and the histologic grading of the tumor or a delay in treatment after perforation showed no correlation with long-term survival[10]. Earlier, in 1997, Adachi et al. reviewed 155 cases of perforated gastric cancer collected from the Japanese literature finding that infiltrative gross type of the tumor, presence of serosal invasion, presence of lymph node metastasis, stage III-IV and curability of the tumor were the only negative prognostic factors influencing the 5-years survival rate, while age, sex, location, histologic type and type of lymph node dissection were not found to be significantly related to the long term survival[1]. In another study of Gertsch et al., the Authors compared three groups of patients with perforated, bleeding and non-complicated gastric cancer, finding that perforation, as well as bleeding, does not significantly affect long term survival after gastrectomy[23].

Treatment of choice is still debated. Table 3 shows the results of our research in the International English literature. From the first study of Aird[24] in 1935 until the early 1980's we found how the most frequent type of operation performed for perforated gastric cancer was the simple closure or the omental patch, sometimes associated with gastroenteroanastomosis. In these papers is also shown the high surgery-related mortality of this type of surgery, nevertheless surgeons seemed to prefer simple repair, probably because malignant gastric perforation, with consequent peritoneal dissemination of tumor cells, was generally thought to be always a manifestation of terminal disease. Of course, the high mortality of simple closure is also due to the different kind of patients who undergo this type of minimal surgery: this approach is usually preferred for minimal therapy in frail patients or in advanced unresectable tumors. Therefore over the years the resection rate has been increasing and the overall mortality rate has been decreasing. In 2002 Lehnert et al.[9] proposed the two-stage radical gastrectomy as the treatment of choice in the majority of patients with perforated gastric cancer: this approach aims to avoid major surgical procedures in emergency performing a first-step simple closure or a gastric resection and later, a secondary elective gastrectomy with oncological radicality intent. This kind of approach has been approved by Ozmen et al.[25] who found that preoperative shock is a negative prognostic factor influencing surgery-related mortality.

**Table 3:** Published series of patients with perforated gastric cancer.

						Mortality (%)		
Reference	N° patients	Incidence (%)	Preoperative diagnosis (%)	N° Repair surgery	N° Gastrectomy	Repair	Gastrectomy	Survival data
Aird 1935[24]*	38	-	7.5	31	7	22 (71)	0	-
McNealy 1938[4]*	63	4.0	33.8	47	7	39 (82)	2 (29)	-
Casberg 1940[31]	5	2.4	0	5	0	4(80)	-	-
Bisgard 1945[5]*	115	2.8–6.0	3.2	80	15	59(74)	2(13)	-
Larmi 1962[13]	19	3.0	42.1	16	4	8(50)	0	Survival range in resected cases 18–42 months
Wilson 1966[12]	14	1.2	30.8	5	5	0	0	Survival range of patients with R_0 _resection, 15–41 months; with R_1_-R_2 _4–15 months
Cortese 1972[11]	13	0.6	40.0	11	2	3(27)	0	Survival range of patients with R_0 _resection, 14–108 months; with R_1_-R_2 _2 months
Stechenberg 1981[3]	9	3.9	0	7	2	2(29)	0	Mean survival, 5 months (range 1–18)
Siegert 1982[32]	4	2.3	25	0	4	-	0	Range of survival, 1–18 months
Miura 1985[20]	9	0.6	33.3	1	8	-	-	Median survival, 108 months (range, 4–144)
Gertsch 1995[10]	34	-	29.4	4	30	2(50)	5(17)	Median survival stage I 50 months; III, 17 months; IV 4 months
Adachi 1997[1]*	155	0.5–3.6	34.7	27	128	19(70)	9(7)	5-years survival stage I-II, 76%; III-IV, 19%
Lehnert 2000[9]	23	1.8	39.1	12^†^	11	1(8)	2(18)	5-years survival R_0_, 50%; 2-years survival R_1_-R_2_, 9%
Kasakura 2002[2]	16	0.7	31.2	2^‡^	14	1(50)	1(7)	Median survival stage I-II 75 months; III-IV, 4.8 months
Ozmen 2002[25]	14	3.0	35.7	3^§^	11^‡^	1(33)	4(36)	-
IRGGC 2005	10	0.4	30.0	4	6	3(75)	1(17)	See text and Table 2

* Collected series; ^† ^5 patients underwent secondary radical gastrectomy; ^‡ ^1 patient underwent secondary radical gastrectomy; ^§ ^2 patients underwent secondary radical gastrectomy.

## Conclusion

**Figure 1:**
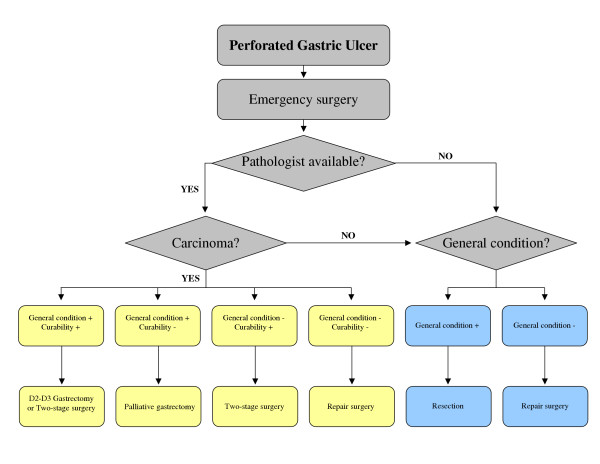
Decisional flow-chart for perforated gastric cancer. General condition includes 3 factors: haemodynamics, gravity of peritonitis and comorbidities.

From the the personal experience of the IRGGC and from the studies reported in the literature we tried to make the point for the treatment of choice of perforated gastric carcinoma. Perforated gastric carcinoma is not to be considered as a unique disease, but the surgeon should consider the single elements that compose every peculiar clinical case. The treatment of the peritonitis would require a minimal surgery in order to avoid major procedures in an emergency situation; on the other hand the treatment of gastric cancer would require an oncological-oriented surgery in order to satisfy oncological radicality criteria. These two aims are not always compatible in a single emergency surgical treatment. The most important factors to be recalled in the management of a patient with histological diagnosis of perforated gastric carcinoma are: 1) the presence of preoperative shock[26]; 2) the gravity of peritonitis; 3) the curability of the neoplasm; 4) eventual comorbidities of the patient. If we add together points 1, 2 and 4 considering them as the general condition of the patient, we may identify four classes of patients with different options for surgical treatment (Figure 1). If a patient has a curable tumor and acceptable general condition, for example no signs of shock, localized peritonitis and no comorbidities, the treatment of choice seems to be radical total or subtotal gastrectomy with associated D2 or D3 lymphadenectomy or, for a less aggressive approach, two-stage radical gastrectomy. When general condition is good but the tumor is at an advanced stage with no possibility of R_0 _resection, a palliative gastrectomy, if technically possible, is recommended considering the minor surgery-related mortality[27]. Two-stage radical gastrectomy seems to find its peculiar indication when general condition is poor but a curative resection is possible, even though this approach was never chosen in our experience. Simple repair or omental patch are reserved only for those patients with advanced stage disease and whose general condition is poor. If a pathologist is not available and histologic examination is not possible during surgery, we suggest to perform a gastric resection, since for perforated peptic ulcer too the treatment of choice is resection both for the better morbility and the lower rate of recurrence [28-30]; only intraoperative hemodynamic instability should limit operative selection to a faster procedure. In both cases when the postoperative histologic examination would assess the malignancy of the ulcer a secondary radical gastrectomy is mandatory.

## Competing interests

The author(s) declare that they have no competing interests.

## Authors' contributions

FR: conceived of the study and participated in the design of the study

SR: participated in the design of the study and drafted the manuscript

DM: participated in the design of the study and performed the statistical analysis

GDM: participated in the design of the study

CP: participated in the design of the study

PM: participated in the design of the study

GC: participated in the design of the study and helped to draft the manuscript

EP: coordinated the study
